# Contrast gain control occurs independently of both parvalbumin-positive interneuron activity and shunting inhibition in auditory cortex

**DOI:** 10.1152/jn.00587.2019

**Published:** 2020-03-18

**Authors:** James E. Cooke, Martin C. Kahn, Edward O. Mann, Andrew J. King, Jan W. H. Schnupp, Ben D. B. Willmore

**Affiliations:** ^1^Department of Physiology, Anatomy and Genetics, University of Oxford, Oxford, United Kingdom; ^2^University College London, London, United Kingdom; ^3^Department of Biomedical Sciences, City University of Hong Kong, Hong Kong

**Keywords:** auditory cortex, circuit mechanisms, contrast gain control, parvalbumin-positive interneurons, shunting inhibition

## Abstract

Contrast gain control is the systematic adjustment of neuronal gain in response to the contrast of sensory input. It is widely observed in sensory cortical areas and has been proposed to be a canonical neuronal computation. Here, we investigated whether shunting inhibition from parvalbumin-positive interneurons—a mechanism involved in gain control in visual cortex—also underlies contrast gain control in auditory cortex. First, we performed extracellular recordings in the auditory cortex of anesthetized male mice and optogenetically manipulated the activity of parvalbumin-positive interneurons while varying the contrast of the sensory input. We found that both activation and suppression of parvalbumin interneuron activity altered the overall gain of cortical neurons. However, despite these changes in overall gain, we found that manipulating parvalbumin interneuron activity did not alter the strength of contrast gain control in auditory cortex. Furthermore, parvalbumin-positive interneurons did not show increases in activity in response to high-contrast stimulation, which would be expected if they drive contrast gain control. Finally, we performed in vivo whole-cell recordings in auditory cortical neurons during high- and low-contrast stimulation and found that no increase in membrane conductance was observed during high-contrast stimulation. Taken together, these findings indicate that while parvalbumin-positive interneuron activity modulates the overall gain of auditory cortical responses, other mechanisms are primarily responsible for contrast gain control in this cortical area.

**NEW & NOTEWORTHY** We investigated whether contrast gain control is mediated by shunting inhibition from parvalbumin-positive interneurons in auditory cortex. We performed extracellular and intracellular recordings in mouse auditory cortex while presenting sensory stimuli with varying contrasts and manipulated parvalbumin-positive interneuron activity using optogenetics. We show that while parvalbumin-positive interneuron activity modulates the gain of cortical responses, this activity is not the primary mechanism for contrast gain control in auditory cortex.

## INTRODUCTION

Contrast gain control is a common computational principle in multiple sensory systems (Carandini and Heeger 2012). As the contrast of sensory inputs increases, the gain of neural responses in visual (Carandini and Heeger 1994; Carandini et al. 1997; Heeger 1992; Heeger et al. 1996) and auditory (Rabinowitz et al. 2011, 2012) cortices is reduced. This helps to create sensory representations that are invariant to factors such as changes in illumination or background noise. Contrast adaptation has also been shown to affect perceptual thresholds (Greenlee and Heitger 1988; Lohse et al. 2020).

Since the computation of contrast gain control may be similar across cortical areas, it is also possible that the neural circuits that implement contrast gain control may be conserved between areas. Indeed, the similarity of circuit architecture across cortical areas raises the possibility of a common mechanism (Douglas and Martin 1991). In primary visual cortex (V1), gain control has been attributed to a combination of inhibition from parvalbumin (PV)-positive interneurons (PVIs; Atallah et al. 2012; Wilson et al. 2012), shunting inhibition (Carandini and Heeger 1994; Carandini et al. 1997), fluctuations in membrane potential (Finn et al. 2007), and synaptic depression. Each of these mechanisms could also contribute to contrast gain control in auditory cortex.

Optogenetic activation of PVI firing reduces the gain of spiking responses in V1, whereas reducing PVI firing has been found to increase gain (Atallah et al. 2012; Wilson et al. 2012). These manipulations left tuning bandwidth and the orientation preferences of V1 pyramidal cells unchanged, indicating that the principal effect of PVI activity was to modulate neuronal gain. PVI inhibition is also a plausible candidate mechanism for contrast gain control in auditory cortex. PVIs tend to have larger sound-level dynamic ranges and lower response gains and are more likely to exhibit monotonic response-level functions than PV-negative neurons (Moore and Wehr 2013). Thus, their responses appear to be more linear with respect to stimulus level than those of pyramidal cell responses, a characteristic that may make them well suited for implementing stimulus-driven gain control in auditory cortex. PVIs in auditory cortex also show frequency tuning that may be matched to the local population (Moore and Wehr 2013), a feature consistent with the finding that auditory cortical contrast gain control predominantly depends on contrast within the receptive field of the neuron (Rabinowitz et al. 2012). Should PVI inhibition underlie contrast gain control in auditory cortex, this would suggest that it may provide a canonical mechanism for gain control across sensory cortical areas.

PVIs are thought to alter neuronal gain by modulating input conductance, a process known as shunting inhibition (Borg-Graham et al. 1998; Frégnac et al. 2003). This has a scaling effect on the amplitude of postsynaptic potentials (PSPs), with increases in conductance reducing PSP size. In keeping with the hypothesis that shunting inhibition contributes to gain control, the input conductance of V1 neurons has been found to increase by up to 300% from baseline during stimulation with high-contrast gratings (Anderson et al. 2000; Monier et al. 2003).

Another candidate mechanism for contrast gain control involves contrast-dependent fluctuations in membrane potential. With no synaptic noise present, the relationship between membrane potential and the firing rate of cortical pyramidal cells is captured by a linear threshold function. In vivo, membrane potential variability smooths this relationship so that it approximates a power law function (Miller and Troyer 2002), which can increase gain by increasing spiking probability for a given mean membrane potential. In V1, reductions in this variability with increasing contrast reduce the gain of cortical responses, contributing to gain control (Carandini 2004; Finn et al. 2007; Hansel and van Vreeswijk 2002). It is unknown whether a similar mechanism is found in auditory cortex.

In this study, we investigated whether shunting inhibition from PVIs or fluctuations in membrane potential contribute mechanistically to contrast gain control in mouse auditory cortex. We found that optogenetically manipulating PVI activity modulated response gain but had much smaller effects on contrast gain control. Furthermore, PVI activity and neuronal input conductance were not modulated by stimulus contrast. We also found that stimulus contrast had no effect on membrane potential fluctuations. These findings indicate that mechanisms implicated in gain control in visual cortex do not contribute to contrast gain control in auditory cortex.

## MATERIALS AND METHODS

A total of 38 male mice between 4 and 12 wk old were used in this study. The strains used are given in the following sections. All procedures received approval from the Oxford University Animal Care and Ethical Review Committee and were licensed by the UK Home Office.

We performed all extracellular and intracellular recordings from the auditory cortex of anesthetized mice. The anesthetized preparation was deemed appropriate for these experiments as contrast gain control in the auditory cortex (Rabinowitz et al. 2011) and midbrain (Lohse et al. 2020) has been found to be unaffected by anesthesia. Similarly, research into the role of PV interneuron activity is routinely investigated in both awake (e.g., Natan et al. 2017) and anesthetized (e.g., Atallah et al. 2012) preparations, and no difference between these conditions was reported in experiments in which the effects of optogenetic activation of PV interneurons on activity in auditory cortex was examined (Blackwell et al. 2020). Finally, anesthesia was a constant factor across conditions and so should not have been a relevant variable for the between-condition changes observed here. Anesthesia was induced through an intraperitoneal (i.p.) injection of 0.1 mg/kg ketamine (Narketan, Chassot AG, Belp, Bern, Switzerland) and 0.25 mg/kg medetomidine (Domitor, Pfizer). 0.1 mg atropine (Atrocare, Animal Care Ltd.) and 0.01 mg dexamethasone (Dexadreson, MSD Animal Health) per animal were delivered subcutaneously to reduce lung secretions and cerebral edema, respectively. Anesthesia was maintained via i.p. infusion of 50 mg·kg^−1^·h^−1^ ketamine and 0.06 mg·kg^−1^·h^−1^ medetomidine. We monitored core body temperature throughout the experiment and maintained it at 37.5 ± 0.5°C. We assessed anesthesia levels regularly via the pedal withdrawal (paw pinch) reflex and any signs of whisker movement. We anesthetized the scalp locally using 0.1 g Marcain (AstraZenica) before exposing the left temporal and parietal bones of the skull. We anesthetized the left temporal muscle locally using 0.1 mg Marcain and subsequently retracted the muscle, using diathermy to cauterize any bleeding. The skull was then fixed to a head plate using a combination of tissue adhesive (Vetbond, Santa Cruz Animal Health) and dental acrylic.

We identified the location of auditory cortex using cranial landmarks and local vasculature. Craniotomies extended from the lambdoid suture at the most caudal extent to 1 mm rostral of the point at which the squamosal suture crosses the temporal ridge. In the dorsal-ventral axis, the craniotomy extended from 2 mm dorsal of the temporal line to the squamosal suture at the most ventral extent. Recording sites close to the largest vessel in this area, oriented along the dorsal-ventral axis, were consistently responsive to auditory stimuli. We used the frequency tuning of auditory responses to confirm that recordings were localized to auditory cortex. However, it was not possible to map specific auditory cortical fields in these experiments, so auditory cortex recordings likely include responses from secondary as well as primary cortical areas.

### Extracellular Recordings

We made extracellular recordings from the auditory cortex of mice between 8 and 12 wk of age, using silicon multielectrodes (NeuroNexus). There were two groups of mice, in which PVI activity was manipulated via either archearhodopsin (Arch) or channelrhodopsin.

#### Arch experiments.

In these experiments (*n* = 8 mice), we aimed to suppress PVI activity. To achieve this, we crossed homozygous PV^cre^ (Jax No. 008069, Hippenmeyer et al. 2005) with homozygous Ai35D mice (Jax No. 012735, Madisen et al. 2012), which express Arch (Chow et al. 2010) and green fluorescent protein (GFP) in a cre-dependent manner. PV^cre^ x Ai35D offspring therefore express Arch and GFP in PVIs. Multiunit activity was recorded from the auditory cortices of the offspring using 1×32 linear multielectrodes (17 penetrations).

#### Channelrhodopsin experiments.

In another set of mice (*n* = 20), we aimed to stimulate PVI activity. We targeted expression of channelrhodopsin (ChR2, Boyden et al. 2005) and enhanced yellow fluorescent protein (eYFP) to PVIs to increase PVI activity during recordings. PV^cre^ mice were anesthetized using i.p. 0.05 mg/kg fentanyl (Sublimaze, Janssen Ltd.), 0.5 mg/kg medetomidine (Domitor, Pfizer), and 5 mg/kg midazolam (Hypnovel, Roche Products Limited). The anesthetized animals then received stereotaxic injections of 500 nL AAV-EF1a-DIO-hChR2(H134R)-EYFP-WPRE-pA serotype 2 (UNC Vector Core) in auditory cortex. The virus was injected 3–5 wk before terminal recordings, to allow for expression of ChR2 and EYFP in PVIs.

#### Multiunit analysis.

We recorded multiunit activity (MUA) in 14 of these mice using 1×32 linear multielectrodes (29 penetrations) and performed single-unit recordings in another 6 mice using 64-channel Buzsaki probes (NeuroNexus, 6 penetrations). We identified single units manually following spike sorting using KlustaKwik (Rossant et al. 2016). We used an analog measure of the MUA recorded on each channel, which involved measuring the voltage signal power in the frequency range occupied by spiking activity by band-pass filtering the recorded signals (Chung et al. 1987; Choi et al. 2010; Kayser et al. 2007; King and Carlile 1994; Schnupp et al. 2015; Schroeder et al. 1998). For each channel, we band-pass filtered the recorded voltage signal between 300 and 6,000 Hz. We then low-pass filtered the full-wave rectified signal below 6,000 Hz and downsampled it to a sample rate of 12,000 Hz. We also extracted local field potentials by low-pass filtering the recorded signals below 300 Hz using a digital 8th order Chebyshev Type I filter.

#### Light stimulation.

We manipulated PVI activity by exposing auditory cortex to either amber light (595 nm; Arch experiments only) or blue light (450 nm, channelrhodopsin experiments only), delivered by an LED (Doric Lenses Inc.) coupled to a 0.55-mm-diameter fiber-optic cable. We positioned the fiber-optic cable over the recording site using a micromanipulator, with the tip <1 mm above the surface of the cortex. The power output of the LED was calibrated for each penetration. We calibrated the strength of the optogenetic manipulations by adjusting the light intensity to produce a 5% difference in the peak evoked firing rate to noise burst stimuli. We performed post hoc quantification of the strength of the optogenetic manipulations only for multiunits (MUs) that were included in subsequent analysis, after meeting the selection criterion of having a predictive spectrotemporal receptive field (STRF). We assigned recordings to cortical layers using current source density (CSD) profiles of noise responses. The highest powers used were 1.2 mW (amber) and 3.6 mW (blue).

Continuous LED illumination of the cortex occurred on a pseudo-randomly selected half of trials, both for noise burst stimuli and for dynamic random chords (DRCs) (see *Dynamic Random Chords*). For the noise bursts, light onset occurred 250 ms before sound onset and lasted for 750 ms. For DRCs, light onset was coincident with stimulus onset and lasted for the entire duration of the stimulus (40 s).

#### Stimulus presentation.

In all animals, we made recordings in the left auditory cortex and delivered stimuli to the contralateral ear. All stimuli had a mean sound level of 80 dB SPL. We presented stimuli using an Ultrasonic Dynamic Speaker (Avisoft Bioacoustics, Glienicke, Germany), modified for monaural in-ear delivery. The speaker was driven using a TDT RX6 multifunction processor (Tucker-Davis Technologies) at a sample rate of 200 kHz. We calibrated stimuli across the frequency range of 1–64 kHz using a Brüel and Kjær Type 4138 1/8th inch pressure-field microphone to assess the response of the speaker across this range. We then created an inverse filter based on this response, which was used to produce a flat frequency response within 3 dB. We controlled stimulus presentation and data acquisition using in-house software (Benware; https://github.com/beniamino38/benware). Subsequent analysis was carried out in MATLAB (The MathWorks, Natick, MA).

#### Noise bursts.

Bursts of broadband noise (50-ms duration) were delivered to test for acoustic responses at the recording sites and to verify the effectiveness of the optogenetic manipulations on these responses. We constructed peristimulus time histograms (PSTHs) of multiunit (MU) responses by binning spike times after stimulus onset into 25-ms time bins. The first two bins following light onset were removed to avoid light artifacts. For both “light-on” and “light-off” conditions, the bin within 150 ms of noise onset with the highest MUA signal amplitude was taken as the peak response. We used the difference in peak firing rate between light-on and light-off conditions to quantify the effect of the optogenetic manipulations. Each penetration showed noise-evoked responses on multiple channels. For the Arch-expressing mice, 85% of MUs recorded showed noise-evoked responses, defined as at least a doubling of baseline firing in response to noise onset. For the ChR2-expressing mice, 40% of MUs recorded showed noise-evoked responses, according to this criterion. Noise responsiveness was only used as a selection criterion when assessing the strength of optogenetic effects. Recordings from ChR2-transfected mice showed fewer noise-responsive neurons, which could be due to the additional injection surgery before recording. Furthermore, the 3-wk postinjection period required for viral expression in this group resulted in the ChrR2 mice typically being several weeks older than Arch mice, which may have also contributed to this difference. LED illumination of the cortex occurred on a pseudo-randomly selected half of trials. Light onset occurred 250 ms before sound onset and lasted for 750 ms.

### Dynamic Random Chords

As in previous work (Cooke et al. 2018), we used dynamic random chord (DRC) stimuli to deliver sounds with adjustable spectrotemporal contrast. Each DRC is a series of chords, where each chord is a superposition of pure tones. The duration of individual chords and the frequencies of the tones were fixed. However, the sound level (dB SPL) of each tone varied randomly from chord to chord according to a uniform probability distribution. All DRCs in this study consisted of 25 superposed pure tones, with frequencies from 1 kHz to 64 kHz in ¼-octave steps. Each chord lasted 25 ms, including 5-ms linear onset and offset ramps.

To vary the contrast of the DRCs, we used different uniform probability distributions for the sound level of the tones. The mean of the distributions was fixed at 40 dB SPL. In the low-contrast condition, the distribution had a range of 20 dB (σ_L_ = 6.2 dB, *c* = σ_P_/μ_P_ = 0.7); in the high-contrast condition, the range was 40 dB (σ_L_ = 12.0 dB, *c* = σ_P_/μ_P_ = 1.2). Each DRC sequence consisted of 1,600 chords, lasted 40 s in total, and was presented three times. For each condition, four DRC stimuli were used and each DRC was presented three times. Each lasted 40 s, resulting in 480 s of DRC data for model fitting for each condition.

### Spectrotemporal Receptive Fields

DRCs have previously been used to measure the spectrotemporal tuning of auditory neurons (Ahrens et al. 2008; Bitterman et al. 2008; Christianson et al. 2008; deCharms et al. 1998; Linden et al. 2003; Rutkowski et al. 2002; Sahani and Linden 2003; Schnupp et al. 2001) by constructing spectrotemporal receptive fields. Neurons in the auditory system typically respond preferentially to particular frequencies, as well as to temporal modulations. STRFs are models of neuronal responses that are capable of capturing these features. We estimated STRFs using a space-time separable model (Ahrens et al. 2008), to reduce the number of parameters and therefore reduce overfitting. This involves fitting a frequency kernel, *k_f_*, and a history kernel, *k_h_*, and computing the outer product of these two kernels, *k_fh_*:

$$k_{fh}=k_{f}⊗k_{h}$$

We fitted each kernel in turn by least-squares linear regression while holding the other kernel fixed, and repeating this procedure to convergence. We then measured the best frequency (BF), spectral bandwidth, and temporal integration window of each STRF. The BF was defined as the frequency bin in the frequency kernel with the largest coefficient. The spectral bandwidth was measured as the width of the tuning curve around this peak response in the frequency kernel at half of its amplitude. The temporal integration window was measured as the width of the tuning curve surrounding the peak coefficient observed in the first 100 ms of the history kernel. The measurement was taken at 50% of the amplitude of the peak coefficient.

### Multiunit Selection Criterion

To be included in further analysis, we required MUs to meet the selection criterion of having a predictive STRF. We fitted STRFs to 90% of responses to DRCs during light-off control trials and examined their predictive performance by using the STRFs to predict responses to the remaining 10% of DRCs. In previous work all predictive STRFs were included in further analysis no matter how weakly predicted they may have been (Rabinowitz et al. 2011). Here, however, we considered units unpredictive if the correlation coefficient (CC) between the predicted and actual response was smaller than 0.04 and excluded them from further analysis.

### Estimation of Gain Changes

For each MU, we quantified baseline activity under each condition as the 5th percentile of the response of the MUA (*R*_min_) and the maximum response as the 95th percentile (*R*_max_). We quantified changes in *R*_max_ and *R*_min_ by taking the ratio of each of these quantities under light-on ($R_{\text{max}}^{\text{on}}$*)* and light-off ($R_{\text{min}}^{\text{off}}$) conditions. Subtractive or additive changes in the offset of responses (*R*_offset_) produced by changes in PVI activity were quantified by taking the difference between *R*_min_ under light-on ($R_{\text{min}}^{\text{on}}$) and light-off conditions ($R_{\text{min}}^{\text{off}}$) and normalizing this by the full range of responses:

$$R_{\text{offset}}=\frac{R_{\text{min}}^{\text{on}}-R_{\text{min}}^{\text{off}}}{R_{\text{max}}-R_{\text{min}}}$$

For each combination of optogenetic (light on versus light off) and stimulus (high versus low-contrast) conditions, we quantified the range of responses, *S*, as the difference between *R*_max_ and *R*_min_, i.e.:

$$S=R_{\text{max}}-R_{\text{min}}$$

giving the range in each of the four conditions: $S_{\text{high}}^{\text{off}}$, $S_{\text{low}}^{\text{off}}$, $S_{\text{high}}^{\text{on}}$, $S_{\text{low}}^{\text{on}}$.

We then calculated the relative strength of gain control in light-on and light-off conditions. This is a measure of the extent to which optogenetic manipulation of PVI activity affects contrast gain control, where a value of 1 indicates no effect.

$$G_{\text{relative}}=\frac{S_{\text{low}}^{\text{on}}/S_{\text{high}}^{\text{on}}}{S_{\text{low}}^{\text{off}}/S_{\text{high}}^{\text{off}}}$$

In addition to this nonparametric analysis of gain, we also performed a parametric analysis similar to that described in Rabinowitz et al. (2011). Fitting the parametric model to each condition individually proved challenging for some MUs in this data set, and so the parametric fits were obtained by fitting a single model to all data from each optogenetic condition. In this model, only the gain and *x*-offset parameters were allowed to vary between contrast conditions. The parametric analysis gave similar estimates of changes in the strength of contrast gain control. However, it does not provide a direct method to estimate the changes in overall gain between optogenetic conditions, and so we have presented the nonparametric analysis here.

### Histology

We confirmed expression of viral constructs in PVIs by histological analysis. Following completion of terminal experiments, mice were perfused transcardially with 4% paraformaldehyde in phosphate-buffered saline (PBS). We removed the brain from the skull and immersed it in 4% paraformaldehyde for a minimum of 24 h. After fixation was complete, 50-μm sections were cut using a cryotome. PVIs were identified by immunohistochemical labeling with a fluorescent marker. We rinsed sections with 0.25% Triton in PBS before incubating them in a 10% donkey serum solution for 1 h to block background labeling. They were then incubated in a 1/4,000 PV goat antibody (Abcam, Cambridge, MA, ab32895) PBS solution with 3% donkey serum for 48 h at 4°C. We next rinsed the sections in 0.25% PBS Triton before incubating them in a secondary red fluorescent antibody in 0.25% PBS Triton (1/1,000 donkey anti-goat IgG Cy3, Abcam, Cambridge, MA, ab6949). Finally, we rinsed sections in PBS before mounting them on glass slides and coverslipping with VectaShield HardSet mounting medium with DAPI (VectorLabs, Burlingame, CA).

### Intracellular Recordings

We performed blind whole-cell patch recordings from the auditory cortex of eight C57BL/6 mice, between the ages of 4 and 10 wk. We removed the dura and applied saline to the exposed cortex to avoid dehydration. We inserted silver-plated reference and ground wires into frontal and parietal cortices, respectively, through two small craniotomies, and fixed them in place using dental acrylic.

We oriented electrodes 45° to the cortical surface and advanced them to depths of between 300 and 700 μm to record from the middle layers of the cortex, including lower layer 2/3, 4, and 5. We used conventional, low-resistance (4–7 MΩ) patch pipettes made from borosilicate glass (World Precision Instruments). The intracellular solution consisted of (in mM): 110 K-gluconate, 40 HEPES, 2 ATP-Mg, 0.3 GTP, 4 NaCl, and 4 mg/ml biocytin, pH 7.2–7.3. We used a Multiclamp 700B amplifier (Molecular Devices) to amplify intracellular recordings. As with the extracellular recordings, we used a TDT RZ2 digital signal processor to digitize the data.

Unlike extracellular recordings, which can be stable over several hours, whole cell recordings can typically only be maintained for tens of minutes. This limitation prevented us from collecting enough data to fit receptive field models as we had done with the extracellular data. The spectrotemporal tuning properties of the intracellularly recorded neurons therefore remain unknown. To probe response properties in neurons with unknown tuning properties, we embedded 50-ms bursts of frozen noise halfway through each DRC. This enabled us to drive responses in neurons irrespective of their individual best frequencies. A similar approach has been used successfully previously to assess the time course of contrast gain control in ferret auditory cortex (Rabinowitz et al. 2011).

Stimuli were presented as described above and consisted of a 1-s DRC that was repeated 11 times, alternating between low-contrast (σ*_L_* = 6.2 dB, *c = *σ*_P_/*μ*_P_* = 0.68) and high-contrast (σ*_h_* = 11.97 dB, *c = *σ*_P_/*μ*_P_* = 1.2) with each repetition while the spectrotemporal pattern of the DRC otherwise remained the same. The 50-ms noise bursts occurred 500 ms into each DRC, replacing the two 25-ms chords that would otherwise have occurred at this time. We constructed such alternating DRC / noise burst sequences using four different random seed tokens for the pseudo-random number sequences that determined the DRCs’ tone levels and the frozen noise burst waveforms in each case. This allowed us to verify that our results generalized to different DRCs and are not specific to one particular DRC pattern or noise burst. We omitted the first low-contrast DRC from the analysis to avoid including onset responses resulting from the transition from silence to auditory stimulation.

To measure the input conductance, *G*, of recorded auditory cortex neurons we injected different levels of current and measured the effect on membrane potential, *V*_m_. The extent to which *V*_m_ changes in response to injected current, *I*_inj_, depends on *G*, as captured in Ohm’s law:

$$\delta V_{\text{m}}=\frac{I_{\text{inj}}}{G}$$

This allowed us to estimate *G* from the recorded change in voltage *V*_m_ in response to different levels of *I*_inj_. The inverse of *G* is the input resistance of the neuron *R*_input_. The resistance of the electrode after the patch is formed, also known as the access resistance and here referred to as *R*_access_, also contributes to the relationship between *V*_m_ and *I*_inj_:

$$\delta V_{\text{m}}=I_{\text{inj}}\cdot \left(R_{\text{access}}+R_{\text{input}}\right)$$

**Fig. 1.**
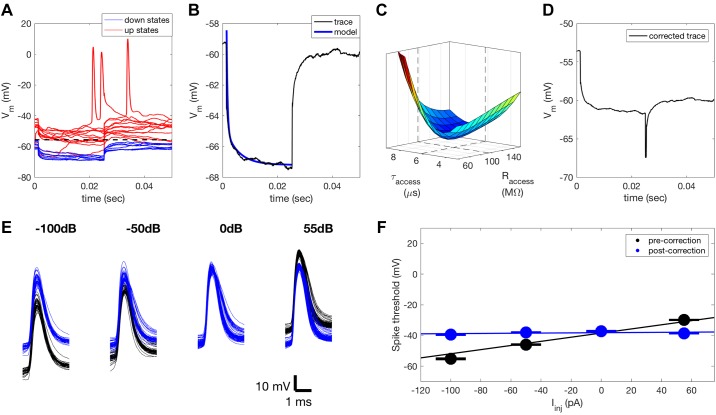
Compensation for access resistance. *A*: we injected a series of 40-pA current pulses into each neuron before each sweep. This allowed us to estimate both *R*_access_ and *R*_input_ from the change in *V*_m_. Cycles of current injection that included an up state (red traces) were identified by crossing a threshold corresponding to the mean of the *V*_m_ distribution. These traces were excluded and the remaining traces (blue) were used to compute the mean *V*_m_ response to the current pulses. *B*: the mean *V*_m_ (blue trace) consists of fast and slow exponential components that correspond to *R*_access_ and *R*_input_, respectively. A double exponential model was fitted to the mean *V*_m_ response to quantify the contribution of *R*_access_ so that it could subsequently be compensated for. *C*: each fit showed a clear minimum in the error function for a range of resistance, *R*_access_, and time constant, τ_access_, values. *D*: estimate of the true *V*_m_ in response to *I*_inj_ following compensation for *R*_access_. *E*: an estimate of the remaining *R*_access_ following compensation was also obtained for each neuron by quantifying the change in spike threshold under different levels of *I*_inj_. Spikes before (black) and after (blue) correction for *R*_access_ using these two methods. *F*: a linear fit (black line) to mean spike threshold for different levels of *I*_inj_ (black circles, standard errors indicated by black lines) can be used to quantify *R*_access_. Error bars appear as a single line due to the minimal variation in threshold between spikes. After correction for *R*_access_, spike threshold is impacted far less by *I*_inj_ (blue), demonstrating that *R*_access_ has successfully been compensated for. *I*_inj_, injected current; *R*_access_, access resistance; *R*_input_, input resistance; *V*_m_, membrane potential.

To accurately estimate stimulus-induced changes in *G* over time, it was first necessary to compensate for *R*_access_ in our recordings. Before each stimulus presentation, current with a square waveform (20 cycles at 20 Hz) was injected into each cell. The recorded change in membrane potential in response to *I*_inj_ consists of two components, a fast change due to *R*_access_ and a slow exponential change due to *R*_input_ (Anderson et al. 2000). To separate these two components, we fitted a double exponential model to the mean recorded change in *V*_m_ over time, *V*(*t*):

$$\delta V\left(t\right)/I_{\text{inj}}=R_{\text{access}}\left[1-\text{exp}\left(-t/\tau_{\text{access}}\right)\right]+R_{\text{input}}\left[1-\text{exp}\left(-t/\tau_{\text{input}}\right)\right]$$

where τ_input_ corresponds to the time constant of the neuron and τ_access_ corresponds to the time constant of the patch electrode. The mean response to *I*_inj_ was calculated from *V*_m_ recordings that did not include an “up state.” Up states are periods of spontaneous depolarization (Destexhe et al. 2003) that can mask the effect of *I*_inj_ on *V*_m_. As this results in a bimodal distribution of *V*_m_ values, up states could be detected by threshold crossing, where the threshold is the mean *V*_m_ recorded during both up and down states (Fig. 1*A*). Sweeps including up states were excluded from further analysis. The model was fitted to the mean *V*_m_ response (Fig. 1*B*). A clear minimum in the mean squared error of the fit for different values of electrode resistance and time constant was consistently observed (Fig. 1*C*). Subtracting the voltage component corresponding to *R*_access_ allowed us to estimate the input resistance (Fig. 1*D*), and hence the membrane conductance, and to observe how this changes as a function of stimulus condition.

To validate this method of *R*_access_ compensation, we examined the effect of current injection on spike threshold both before and after compensation for *R*_access_ (Fig. 1*E*). In keeping with previous studies (Anderson et al. 2000), we assumed that spike threshold would not change dramatically with *I*_inj_. Any change in spike threshold with different levels of *I*_inj_ can therefore be attributed to changes in *V*_m_ resulting from *R*_access_. We detected spikes by crossing of a threshold positioned seven standard deviations above the mean *V*_m_. Spike threshold was detected by finding the inflection point in the spike waveform within 1 ms before threshold crossing. After identifying the mean spike threshold for each *I*_inj_, we fitted a linear model, with a slope that corresponded to *R*_access_ that had not been successfully compensated for (Fig. 1*F*). The slope of this fit was consistently reduced to near 0 following *R*_access_ compensation.

**Fig. 2.**
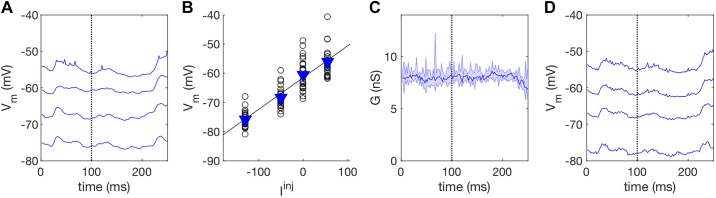
Input conductance estimation. *A*: mean *V*_m_ recorded for four different levels of current injection during dynamic random chord (DRC) and noise stimulation. Input conductance, *G*, was estimated at each time point in the stimulus. The broken line indicates a single time point 100 ms into the stimulus for which we estimated a single value of *G*. *B*: open circles indicate the *V*_m_ recorded at this time point for 25 stimulus repetitions for the four levels of current injection. Blue triangles indicate mean *V*_m_ values plotted in *A*. *G* could be estimated at this time point from these responses by examining the relationship between injected current and recorded *V*_m_ response. This relationship was modeled by fitting a line to these data as Ohm’s law predicts that this relationship should be linear. *C*: the inverse of the slope of the linear fit was used as the estimate for *G* for this time point. *D*: using Ohm’s law, the *V*_m_ could be reconstructed from this estimate for *G* and the known levels of current injection, to validate the estimates. This was necessary as neurons are not perfectly linear devices. *I*^inj^, injected current; *V*_m_, membrane potential.

*G* was estimated by injecting different levels of current into the neuron during sensory stimulation (Fig. 2*A*) as this allowed us to estimate *G* by examining the relationship between injected current and recorded membrane potential. To do this, we fitted a linear model at each time point of the stimulus to the recorded membrane potential responses (Fig. 2*B*). The inverse of slope of the model was used as the estimate for *G* at each time point (Fig. 2*C*). The estimates of *G* over time could then be used to reconstruct the recorded *V*_m_ for different levels of current injection. This allowed us to confirm that for our recording protocol the neuron is responding to current injection according to Ohm’s law and that we are estimating *G* successfully (Fig. 2*D*).

To assess the reliability of the linear model over the recorded range of membrane potentials, we compared the estimated *V*_m_ (Fig. 2*D*) to the recorded *V*_m_ (Fig. 2*A*). This was performed for the entire data set. Predicted values of *V*_m_ captured 97% of the variance in the membrane potential response, indicating that the estimates of *G* were successful and that the relationship between injected current and recorded membrane potential is highly linear.

## RESULTS

### Effects of Optogenetic Manipulations on Evoked Responses

**Fig. 3.**
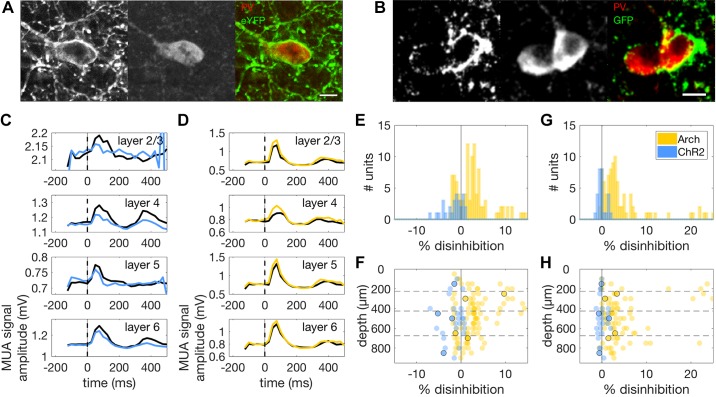
*A*, *left*: Confocal micrograph of a ChR2-eYFP-positive neuron in a section of mouse auditory cortex. *Middle*: PV-positive neuron in a slice labeled with a PV antibody and counterstained with a red fluorescent dye. *Right*: merged image of green (ChR2-eYFP) and red (PV) channels showing coexpression of ChR2-eYFP and PV in the same neuron. Scale bar: 10 μm. *B*: same as in *A* but for coexpression of Arch-GFP and PV in two neurons. *C*: example PSTHs of responses of a MU to 50-ms noise bursts with (blue traces) and without (black traces) optogenetic activation of PVIs. We removed the first 50 ms of the PSTH to exclude photoelectric artifacts from the analysis. All PSTHs show suppression of peak responses under conditions of increased PVI activity (blue), but no suppression of baseline activity (*t* < 0). *D*: PSTHs of noise responses for a MU with (amber traces) and without (black traces) optogenetic suppression of PVIs. As in *C*, control traces with no optogenetic stimulation are shown in black. *E*: histograms showing the effect of PVI activation (blue) and suppression (amber) on peak firing rate in the PSTH for all MUs that showed noise-evoked responses. PVI activation resulted in inhibition of evoked responses, while PVI suppression resulted in disinhibition of responses to noise stimuli. *F*: effects of optogenetic manipulations on evoked neural activity across the depth of cortex. Amber circles correspond to individual MUs under PVI suppression (*n* = 121), while blue circles correspond to individual MUs under PVI activation (*n* = 28). Units whose responses are illustrated in *C* and *D* are indicated here by black circles. PVI activation suppressed noise responses across all cortical layers and did not vary significantly over cortical depth. However, the strength of the disinhibition of noise-evoked responses observed during PVI suppression was found to decrease with cortical depth. *G*: histograms showing the effect of PVI activation (blue) and suppression (amber) on baseline activity in the PSTH for the same MUs as in *E* and *F*. PVI activation had no effect on baseline activity, while suppression resulted in disinhibition of baseline responses. *H*: effects of optogenetic manipulations on baseline activity across the depth of cortex for the same MUs as in *E* and *F*. Units whose responses are illustrated in *C* and *D* are again indicated here by black circles. Effects on baseline activity were consistent across the depth of cortex. Arch, archearhodopsin; ChR2, channelrhodopsin; eYFP, enhanced yellow fluorescent protein; GFP, green fluorescent protein; MU, multiunit; MUA, multiunit activity; PSTH, peristimulus time histogram; PV, parvalbumin; PVI, PV-positive interneurons.

We used two experimental groups of animals in which PVI activity could be up- or downregulated optogenetically. In the first group, ChR2 (Boyden et al. 2005) was expressed in the auditory cortex of PV^cre^ mice by means of a viral vector. The second group was a transgenic strain expressing archearhodopsin (Arch; Chow et al. 2010) in PVIs in auditory cortex. We validated the specificity and efficacy of our optogenetic manipulations in two ways. The AAV-EF1a-DIO-hChR2(H134R)-EYFP-WPRE-pA viral construct was used to target expression of ChR2 and the fluorescent protein EYFP in PVIs. This allowed us to validate the specificity of ChR2-eYFP expression by immunohistochemically staining sections of auditory cortex with a PV antibody and identifying neurons that coexpressed PV. We found expression of ChR2-eYFP in 91% of PV positive neurons (602/661 PV neurons, *n* = 6 mice) with a false positive rate for ChR2-eYFP expression in PV-negative neurons of 1% (31/3943 non-PV neurons, *n* = 6 mice, Fig. 3*A*). Since the Ai35D mouse line we used coexpresses Arch and GFP, we used the same approach to validate endogenous Arch expression. We found that 96% of PV-positive neurons expressed Arch-GFP (569/594, *n* = 6 mice) with a false positive rate for Arch-GFP expression in PV-negative neurons of 3% (92/2914 non-PV neurons, *n* = 6 mice, Fig. 3*B*).

We also validated the optogenetic manipulations by examining their effects on noise responses in auditory cortex. This allowed us to validate the manipulations for each MU included in subsequent analyses (Fig. 3, *C* and *D*). For the ChR2 experiments, 28 MUs met our selection criterion of having a predictive STRF. Optogenetic activation of PVIs significantly inhibited peak responses to noise for these MUs (range of changes: −6.96% to 0.78%, median change: −1.15%; signed-rank test: *P* < 0.001, Fig. 3, *C* and *E*). For the Arch experiments, 121 MUs met the same criterion. For these sites, optogenetic suppression of PVI activity resulted in significant disinhibition of peak MU responses (range of changes: −2.48% to 14.34%, median change: 2.44%; signed-rank test, *P* < 0.001, Fig. 3, *D* and *E*). MUs that met this criterion were included in all subsequent analyses.

To assess the efficacy of the optogenetic manipulation in deeper layers (since optical stimulation was delivered through an LED positioned over the cortical surface), we plotted effect size as a function of cortical depth. We found no significant depth dependence of the inhibition of noise-evoked responses elicited by optogenetic activation of PVIs using ChR2 [median change: layer 2/3 = −1.2%, layer 4 = −2.15%, layer 5 = −0.55%, layer 6 = −3.57%, Fig. 3*F*, blue dots, Kruskal Wallis test: chi-square(3) = 6.65, *P* = 0.919]. However, the disinhibition of evoked responses produced by optogenetic suppression of PVI activity was depth dependent, being significantly stronger in layer 4 than in layer 6 [median change: layer 2/3 = 2.17%, layer 4 = 2.93%, layer 5 = 2.97%, layer 6 = 1.29%, Kruskal Wallis test: chi-square(3) = 11.89, *P* = 0.008; Fig. 3*F*, yellow dots]. This is probably due to reduced light penetration in deep cortical layers, and to weaker expression of endogenous Arch, relative to virally mediated ChR2.

We analyzed the effect of the optogenetic manipulations on spontaneous activity during a silent period. Arch-mediated suppression of PVI activity resulted in significant disinhibition of spontaneous activity (range of changes: −0.58% to 24.92%, median change: 2.74%; signed-rank test: *P* < 0.001) whereas ChR2-mediated activation of PVI activity left spontaneous activity unaffected (range of changes: −1.27% to 1.97%, median change: −0.15%; signed-rank test: *P* = 0.601, Fig. 3*G*). The disinhibition resulting from PVI suppression was observed at all depths [median change: layer 2/3 = 2.07%, layer 4 = 2.93%, layer 5 = 2.51%, layer 6 = 2.92%, Kruskal Wallis test: chi-square(3) = 1.05, *P* = 0.788, Fig. 3*H*, yellow dots]. PVI activation similarly had no layer specific effects on spontaneous activity [median change: layer 2/3 = −0.15%, layer 4 = −0.22%, layer 5 = 0.05%, layer 6 = −0.55%, Kruskal Wallis test: chi-square(3) = 2.51, *P* = 0.474, Fig. 3*H*, blue dots].

We used continuous light stimulation to avoid imposing arbitrary temporal structure on the cortical activity (onset and offset coincident with onset and offset of auditory stimulus). To test whether activity changed over the recording sessions, we recorded single units (*n* = 136) in auditory cortex using Buzsaki style multielectrodes in six mice expressing ChR2 in PVIs. We compared the ratio of firing rates between light conditions in the first half of the stimulus (0–20 s) against the same value for the second half (20–40 s) to investigate whether the effects of stimulation changed throughout this period. The population of 136 single units recorded did not show a significant change in mean firing rate between these two periods (signed-rank, *P* = 0.6753), indicating that the continuous stimulation did not result in any changes in response level over time.

### Suppression of PVI Activity Has Negligible Systematic Effect on STRF Tuning

To investigate the role of PVIs in contrast gain control in mouse auditory cortex, we made extracellular recordings during auditory stimulation, while optogenetically manipulating PVI activity. We measured the effect of the PVI manipulations on the strength of sound-evoked responses, on the tuning of neurons, on their gain, and on contrast gain control itself.

**Fig. 4.**
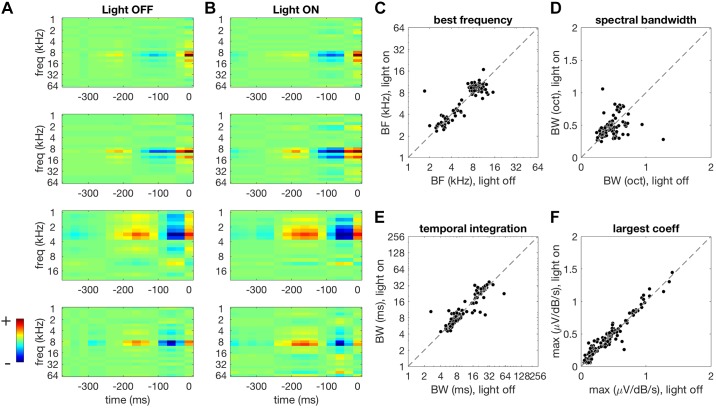
Effect of PVI suppression on STRF shape. *A*: example STRFs estimated for the light-off condition. Spectrotemporal features that increase the MUA amplitude are shown in warmer colors while suppressive features are shown in cooler colors. *B*: STRFs estimated for the same MUAs as in *A*, but under light-on conditions during Arch-mediated PVI suppression. Color scaling is the same as in *A*. This manipulation results in a small increase in peak STRF coefficient. STRFs had similar shapes under light-off and light-on conditions (*A* and *B*). *C*–*F*: comparison of STRF parameters under light-off (abscissa) versus light-on (ordinate) conditions. A small but significant change was observed in best frequency (*C*) under light-on conditions. The bandwidth of frequency tuning (*D*) and temporal integration window (*E*) remained unaffected. The largest STRF coefficient increased during PVI suppression (*F*). Arch, archearhodopsin; BF, best frequency; BW, bandwidth; MUA, multiunit activity; PVI, parvalbumin-positive interneurons; STRF, spectrotemporal receptive fields.

The main aim of the extracellular recordings was to determine the effect of optogenetic PVI manipulations on contrast gain control. However, to compare gain control in the light-on and light-off conditions, we first need to establish whether neuronal tuning is systematically affected by the optogenetic manipulations. If the primary effect of PVI activity is to drive gain changes, then we should not expect optogenetic manipulations of PVI activity to have systematic effects on spectrotemporal tuning. We first investigated this for the Arch data (see materials and methods), where the optogenetic manipulation suppressed the responses of PVIs. We fitted STRFs to MU responses under light-off (Fig. 4*A*) and light-on (Fig. 4*B*) conditions. We then quantified tuning in terms of best frequency (BF), bandwidth of the frequency kernel, temporal integration window, and the largest coefficient in the STRF (a simple measure of gain).

We found that STRF shapes were similar under light-off (Fig. 4*A*) and light-on conditions (Fig. 4*B*). We observed a borderline significant change in mean BF, although median values remained unchanged between light-off and light-on conditions (light-off median: 9.51 kHz, light-on median: 9.51 kHz; light-off mean: 7.61 kHz, light-on mean: 7.81 kHz; signed-rank test, *P* = 0.047; Fig. 4*C*). PVI suppression did not produce a significant change in spectral bandwidth (signed-rank test, *P* = 0.59, Fig. 4*D*) or temporal integration window (signed-rank test, *P* = 0.528, Fig. 4*E*). We did observe, however, a significant increase in the largest STRF coefficient in these MUs during PVI suppression (light-off median: 0.39 V·dB^−1^·s^−1^, light-on median: 0.4V·dB^−1^·s^−1^; signed-rank test, *P* < 0.001, Fig. 4*F*). Overall, these results suggest that PVI suppression has unsystematic, marginally significant effects on STRF tuning but has an appreciable systematic effect on neuronal gain.

**Fig. 5.**
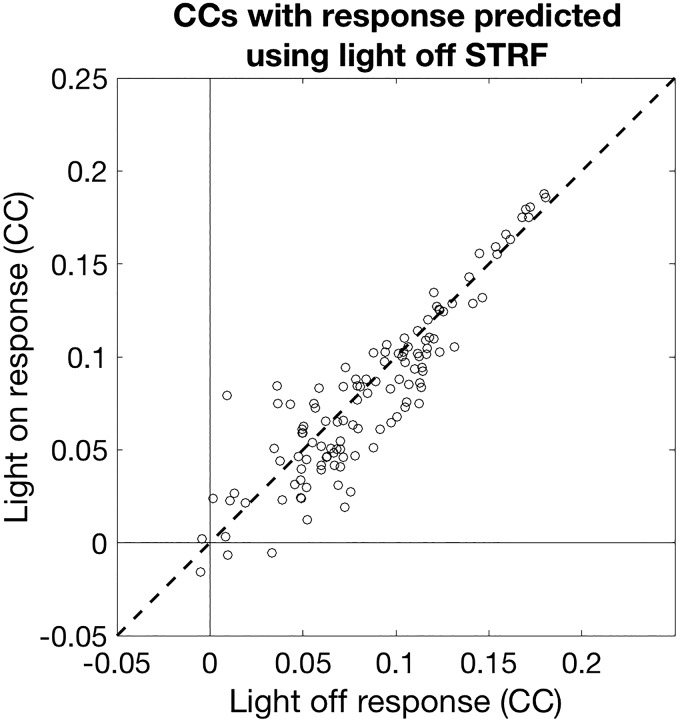
Predictive performance of STRFs across light conditions. Correlation coefficients (CCs) between actual responses and responses predicted by light-off STRFs for both light-off (abscissa) and light-on (ordinate) conditions. STRFs fitted under light-off conditions showed a slight decrement (<2%) in predicting light-on condition responses. These findings indicate that tuning was largely unaffected by manipulation of PVI activity. PVI, parvalbumin-positive interneurons; STRF, spectrotemporal receptive fields.

To assess the importance of the changes we observed in STRF shape, we modeled MU responses to stimuli across light conditions. For each MU, we took the light-off STRF and used it to describe responses in the light-on condition. The only modification we made to the STRF was to incorporate a scale factor (corresponding to a change in gain). We then evaluated the scaled STRF by measuring the correlation between the real and predicted neuronal responses on a held-out data set. We compared these cross-condition predictions with within-condition predictions (where the light-off STRF was tested on held-out data from the light-off condition). Correlations between observed and predicted responses were slightly lower under light-on conditions compared with light-off conditions (light-off median: 0.081, light-on median: 0.079; signed-rank test, *P* < 0.001, Fig. 5). This decrement in performance was only 1.85%, however, indicating that the predictive power of STRFs is largely unchanged when PVI activity is suppressed, which implies that effect of suppressing PVI activity on spectrotemporal tuning of cortical neurons is minimal.

### Suppressing PVI Activity Increases Gain but Leaves Contrast Gain Control Unaffected

For each MU, we quantified changes in baseline activity, maximum response, subtractive/additive changes in the offset of responses and divisive/multiplicative changes in gain between the Arch-mediated PVI suppression condition and a control condition with no optogenetic manipulation (see materials and methods). We also quantified changes in contrast gain control between these two conditions. If PVIs are involved in contrast gain control, optogenetically altering their activity should be expected to affect the magnitude of contrast-dependent gain changes.

**Fig. 6.**
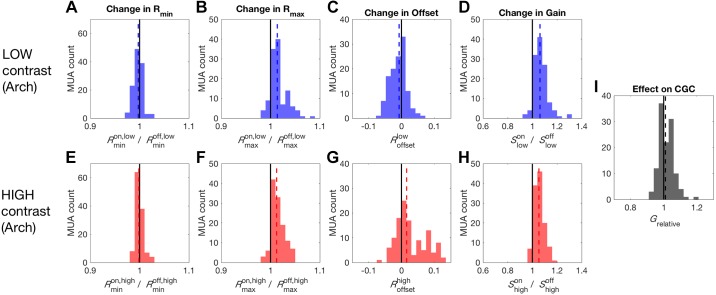
Effect of PVI suppression on auditory cortex responses. *A*: negligible reduction in baseline activity (*R*_min_) produced by PVI suppression during low-contrast stimulation. *B*: a more substantial increase in the maximum response (*R*_max_) was also observed. *C*: a small subtractive change in the offset of responses occurred during PVI suppression. *D*: the asymmetrical effects on *R*_min_ and *R*_max_ led to an increase in gain during PVI suppression. *E*: a small reduction in *R*_min_ was also observed under high-contrast stimulation. *F*: a similar increase in *R*_max_ was observed under high-contrast stimulation. *G*: unlike under low-contrast stimulation, PVI suppression produced a small increase in offset during high-contrast stimulation. *H*: the robust increase in gain observed under low-contrast stimulation was also observed during high-contrast stimulation. *I*: the similarity of the effects on gain under low and high-contrast stimulation resulted in this manipulation leaving the strength of contrast gain control unaffected. Arch, archearhodopsin; MUA, multiunit activity; PVI, parvalbumin-positive interneurons; *R*_min_; 5th percentile of the response of the multiunit activity; *R*_max_, maximum response as the 95th percentile; *S*, range of responses in a particular light condition (“on” vs. “off”) and contrast condition (“low” vs. “high”).

Changes in baseline activity of MUA, *R*_min_*_,_* between conditions were quantified by taking the ratio of the 5th percentile of responses during Arch-mediated PVI suppression over the 5th percentile of responses during the control condition with no optogenetic manipulation. The same was done with the 95th percentile of responses to quantify changes in maximum activity, *R*_max_. For low-contrast stimulation, Arch-mediated PVI suppression produced a reduction in baseline activity during DRC stimulation (*R*_min_ median: 0.997; signed-rank test, *P* = 0.006, Fig. 6*A*) and an increase in maximum activity (*R*_max_ median: 1.014; signed-rank test, *P* < 0.001, Fig. 6*B*), in keeping with the expected disinhibition caused by suppressing inhibitory neural activity. This pattern of changes corresponded to a small subtractive effect on response offset (*R*_offset_ median: −0.007; signed-rank test, *P* < 0.001, Fig. 6*C*) and a clear increase in gain ($S_{\text{low}}^{\text{on}}$/ $S_{\text{low}}^{\text{off}}$ median: 1.062; signed-rank test, *P* < 0.001, Fig. 6*D*). Under high-contrast stimulation, Arch-mediated PVI suppression produced a reduction in baseline activity during DRC stimulation (*R*_min_ median: 0.998; signed-rank test, *P* = 0.046, Fig. 6*E*) and a disinhibitory increase in maximum activity, similar to that observed under low-contrast stimulation (*R*_max_ median: 1.012; signed-rank test, *P* < 0.001, Fig. 6*F*). This also produced a small change in offset (*R*_offset_ median: 0.016; signed-rank test, *P* < 0.001, Fig. 6*G*) and increase in gain ($S_{\text{high}}^{\text{on}}$/$S_{\text{high}}^{\text{off}}$ median: 1.053; signed-rank test, *P* < 0.001, Fig. 6*H*) as observed under low-contrast stimulation. These results indicate that in both high- and low-contrast conditions suppression of PVIs results in a significant increase in gain.

Finally, we assessed the effect of PVI suppression on contrast-dependent gain—i.e., contrast gain control. To do this we measured the relative strength of contrast gain control in light-off and light-on conditions (see materials and methods). We found that the effects of PVI suppression are similar in the low- (Fig. 6, *A*–*D*) and high- (Fig. 6, *E*–*H*) contrast conditions, so that the overall effect of PVI suppression was to leave the strength of contrast gain control unaffected (*G*_relative_ median: 1.012; signed-rank test, *P* = 0.146, Fig. 6*I*).

### PVI Activation Reduces Gain but Leaves Tuning Unaffected

**Fig. 7.**
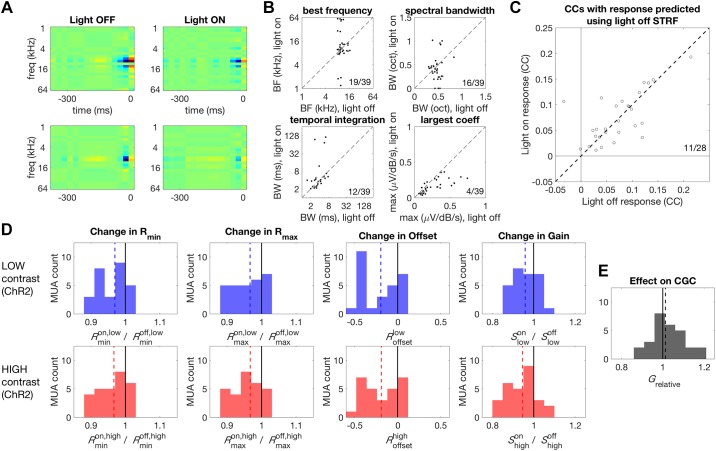
Effect of PVI activation on STRF shape. *A*, *left*: two example STRFs estimated for the light-off condition. Spectrotemporal features that increase the MUA amplitude are shown in warmer colors while suppressive features are shown in cooler colors. *Right*: STRFs estimated for the same MUs as in left panels but under light-on conditions, during ChR2-mediated PVI activation. Color scaling is the same as for the left panels. This manipulation results in suppression of STRF coefficients, but STRF shapes are largely the same under both light-off and light-on conditions. *B*: comparison of STRF parameters under light-off (abscissa) versus light-on (ordinate) conditions. We observed no significant changes in best frequency, spectral bandwidth, or temporal integration window. The largest STRF coefficients were significantly reduced during PVI activation. *C*: predictive performance of STRFs across light conditions. STRFs fitted under light-off conditions predicted responses under light-on and light-off conditions equally well, indicating that tuning was largely unaffected by PVI activation. *D*: effect of PVI activation on auditory cortex responses. A reduction in baseline activity (*R*_min_) was produced by PVI activation during low and high-contrast stimulation (first column). A comparable reduction in the maximum response (*R*_max_) was also observed (second column). PVI activation produced a subtractive change in the offset of responses (third column). A reduction in gain was also observed during PVI activation (fourth column). *E*: PVI activation increased the strength of contrast gain control. BF, best frequency; BW, bandwidth; CCs, correlation coefficients; CGC, contrast gain control; ChR2, channelrhodopsin; *G*_relative_, relative gain change (between conditions); MU, multiunit; MUA, MU activity; PVI, parvalbumin-positive interneurons; *R*_min_; 5th percentile of the response of the multiunit activity; *R*_max_, maximum response as the 95th percentile; *S*, range of responses in a particular light condition (“on” vs. “off”) and contrast condition (“low” vs. “high”); STRF, spectrotemporal receptive fields.

We next investigated the effects of PVI activation on tuning, gain, and contrast gain control using the ChR2 data (see materials and methods). As for the Arch data (above), we first measured the extent to which neuronal tuning was systematically affected by the optogenetic manipulation. We found that, for PVI activation, STRF shapes were generally similar under light-off and light-on conditions (Fig. 7*A*). Activating PVIs with ChR2 had no systematic effect on BF (signed-rank test, *P* = 0.074), bandwidth of the frequency kernel (signed-rank test, *P* = 0.338), or the temporal integration window (signed-rank test, *P* = 0.121, Fig. 7*B*). The magnitude of the largest coefficient in the STRF was, however, significantly reduced under light-on conditions in these MUs (light-off median: 0.16 V·dB^−1^·s^−1^, light-on median: 0.14 V·dB^−1^·s^−1^; signed-rank test, *P* < 0.001, Fig. 7*B*). STRFs that were trained and tested on light-off data predicted responses under light-on and light-off conditions equally well (light-off and light-on median CC: 0.071/0.061; signed-rank test, *P* = 0.665, Fig. 7*C*), indicating the PVI activation did not systematically alter tuning.

Using the nonparametric gain analysis (as described above for Arch data), we found that, for low-contrast stimulation, ChR2-mediated PVI activation produced a significant reduction in baseline (*R*_min_ median: 0.97; signed-rank test, *P* < 0.001) and maximum activity during DRC stimulation (*R*_max_ median: 0.967; signed-rank test, *P* = 0.013, Fig. 7*D*). The comparable size of the reduction in minimum and maximum response resulted in a significant subtractive change in the offset of responses (*R*_offset_ median: −0.196; signed-rank test, *P* < 0.001), in addition to a small reduction in gain ($S_{\text{low}}^{\text{on}}$*/*$S_{\text{low}}^{\text{off}}$ median: 0.958; signed-rank test, *P* = 0.036, Fig. 7*D*). Under high-contrast stimulation, similar reductions in baseline (*R*_min_ median: 0.967; signed-rank test, *P* = 0.004) and maximum activity (*R*_max_ median: 0.967; signed-rank test, *P* < 0.001, Fig. 7*D*) were again observed. This resulted in the same reduction in offset (*R*_offset_ median: −0.19; signed-rank test, *P* < 0.001) and gain ($S_{\text{high}}^{\text{on}}$*/*$S_{\text{high}}^{\text{off}}$ median: 0.946; signed-rank test, *P* < 0.001) observed for low-contrast stimulation (Fig. 7*D*).

Finally, we measured the effect of PVI activation on contrast-dependent gain changes—i.e., contrast gain control. Despite the similarity of the changes in gain induced by PV activation under low and high-contrast stimulation, we found that PVI activation resulted in a small but significant increase in the strength of contrast gain control (*G*_relative_ median: 1.013; signed-rank test, *P* = 0.013, Fig. 7*E*).

Taking the results of PVI suppression and activation together, a consistent picture emerges of the effect of PVI activity on auditory cortical response gain. Suppression and activation of PVIs tends to have opposing effects on neuronal gain, such that higher PVI activity reduces gain while lower PVI activity increases gain. These effects are consistent across stimulus conditions, so that manipulation of PVI activity has only a small effect on contrast gain control.

### PVI Activity Is Not Modulated by Stimulus Contrast

**Fig. 8.**
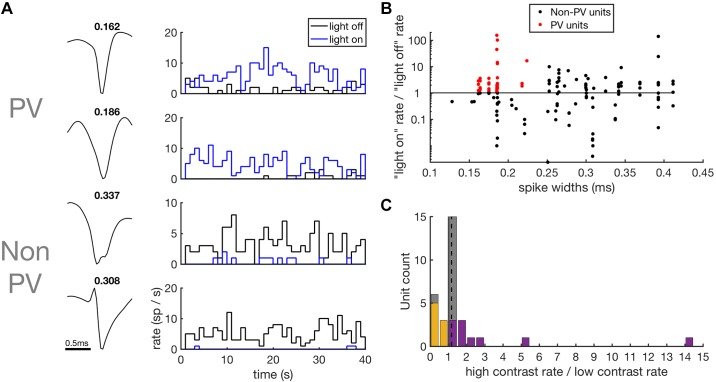
Contrast response of putative PVIs. *A*: spike waveforms and PSTHs of spiking responses of putative PVIs and non-PVIs to high-contrast DRC stimuli with light-off (black) and light-on (blue). Spike widths (ms) are shown above the waveforms. *B*: scatter plot of spike widths (abscissa) against the strength of the optogenetic response (ordinate), measured as the ratio of firing rates during light-on over light-off conditions. To be classified as a putative PVI (red), units were required to show a significant increase in firing rate during light stimulation and to have spike widths of < 0.25 ms. *C*: contrast response of putative PVIs. Histogram of the change in firing rates of putative PVIs in response to a doubling of stimulus contrast. Units that showed a significant increase in firing rate are shown in purple and units that showed a significant decrease are shown in orange. No systematic effect of contrast on firing rates was observed. DRC, dynamic random chord; PSTH, peristimulus time histogram; parvalbumin-positive interneurons; PV, parvalbumin; PVI, parvalbumin-positive interneuron.

We next examined whether PVI activity in the ChR2 animals increases during high-contrast stimulation. To do this, we identified putative PVIs in the single-unit data reported above by examining the extent to which their activity was modulated by exposure to blue light (Fig. 8*A*). The latency of the first spike following light onset is often used for photoidentification of neurons (Lima et al. 2009). This approach is compatible with tungsten electrode recordings, but not with silicon probes recordings, as used here, as large photoelectric artifacts are produced by light onset. These artifacts prevent spike extraction for a period of tens of milliseconds following light onset. This precluded the use of spike-onset latency as a criterion for PVI identification and so different selection criteria were employed here.

Putative PVIs in ChR2 animals were required to show a significant increase in their firing rate under light-on conditions, as assessed by a paired sample *t* test, and to have spike widths of less than 0.25 ms (Cardin et al. 2009; Letzkus et al. 2011; Pi et al. 2013). This second criterion was included to exclude pyramidal cells that may be recruited via disinhibition during light stimulation. Of the 136 single units recorded, 34 passed these criteria and were classified as putative PVIs (Fig. 8*B*). The remaining 102 putative non-PVIs were not included in the following analysis.

We played both high- and low-contrast DRC stimuli under light-off conditions to investigate whether PVIs respond more strongly during high-contrast stimulation. We assessed the contrast response of putative PVIs by taking the ratio of firing rates during high-contrast over low-contrast conditions. As a population, putative PVIs did not show a significant increase in firing rate with increased contrast (high/low-contrast median: 1.18; signed-rank test, *P* = 0.14; Fig. 8*C*). The significance of changes in firing rate with contrast was assessed on a unit-by-unit basis using paired *t* tests. Twelve of the 36 units showed a significant increase in firing rate with contrast, with two of these units only responding during high-contrast stimulation (Fig. 8*C*). Eight units showed a significant decrease in firing rates during high-contrast stimulation (Fig. 8*C*). These results indicate that, as a population, PVIs in auditory cortex are not driven by stimulus contrast.

### Membrane Potential Response Magnitude, but Not Variability, Is Modulated by Stimulus Contrast

**Fig. 9.**
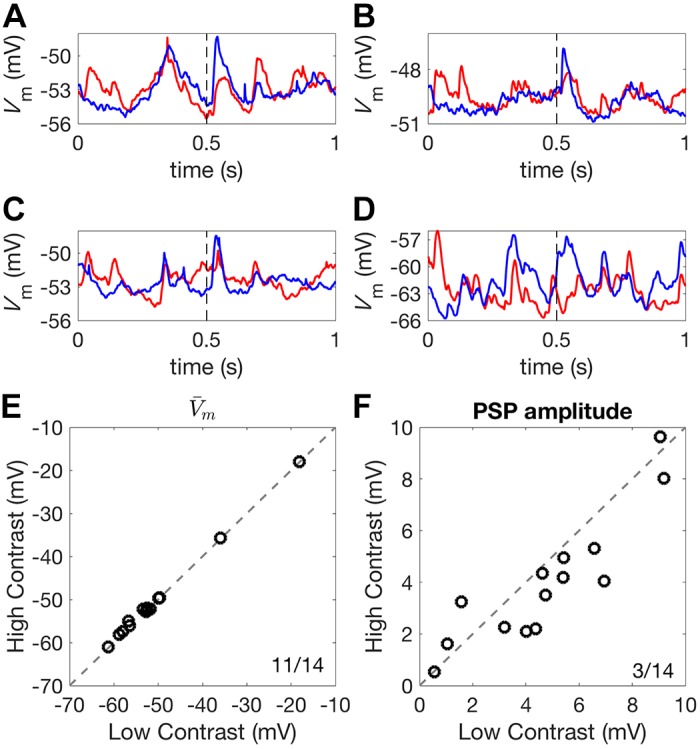
Contrast-dependent membrane potential responses. *A*–*D*: mean *V*_m_ responses to 1-s high-contrast (red) and low-contrast (blue) DRCs for four different auditory cortex neurons. Broken lines indicate the onset of a 50-ms 80 dB SPL noise burst. *E*: mean *V*_m_ showed a very small increase under high-contrast compared with low-contrast stimulation. *F*: the amplitude of the noise-evoked PSP was increased under low-contrast stimulation, relative to high-contrast stimulation. Ratios in the corner of plots indicate the number of neurons above the identity line, *y* = *x*, over the total number of neurons. DRC, dynamic random chord; PSP, postsynaptic potential; *V*_m_, membrane potential.

We performed current clamp recordings from 17 pyramidal cells in the auditory cortex to test whether membrane potential (*V*_m_) responses undergo contrast gain control, as would be predicted if an inhibitory mechanism is involved. No net current was injected into the neuron (0 pA holding current), allowing *V*_m_ responses to DRCs of different contrasts to be recorded (Fig. 9, *A*–*D*).

We first examined whether the mean *V*_m_ responses to DRCs changed with stimulus contrast. We found a small but significant depolarization during high-contrast compared with low-contrast stimulation (low-contrast *V*_m_: −52.62 mV, high-contrast *V*_m_: −52.39 mV; signed-rank test, *P* = 0.009, Fig. 9*E*). We next examined whether noise-evoked PSP amplitudes were modulated by DRC contrast. Noise bursts lasting 50 ms were embedded 500 ms into a 1-s DRC of either low or high contrast. We found that noise-evoked PSP amplitude was suppressed under high-contrast stimulation (low-contrast median: 4.67 mV, high-contrast median: 3.77 mV; signed-rank test, *P* = 0.042, Fig. 9*F*).

**Fig. 10.**
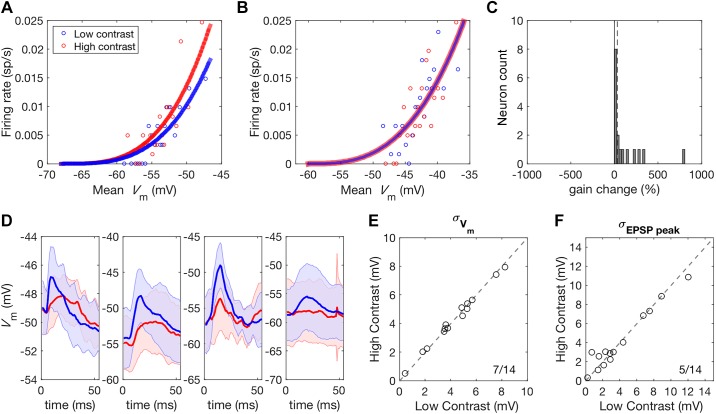
Membrane potential variability in auditory cortex is not contrast dependent. *A*: a power law function was used to map *V*_m_ to firing rate for each unit. Some units, such as this one, showed an increase in the gain of this relationship under high-contrast stimulation. *B*: other units showed no change in gain with stimulus contrast. *C*: histogram of gain changes resulting from increased contrast. No units showed the reduction in gain that would be expected if membrane voltage variance was a determining factor. The broken line corresponds to the median increase in gain with increased contrast. *D*: four representative units showing PSPs evoked by noise stimuli during high (red) and low (blue) contrast stimulation. Shaded areas indicate the standard deviation of *V*_m_ responses across trials. *E*: membrane potential standard deviation during DRC stimulation did not change with stimulus contrast. *F*: similarly, the standard deviation of PSP responses did not show a systematic change with stimulus contrast. Ratios in the corner of plots indicate the number of neurons above the *y* = *x* identity line over the total number of neurons. DRC, dynamic random chord; EPSP, excitatory postsynaptic potential; PSP, postsynaptic potential; σ*_V_*_m_, standard deviation of *V*_m_; *V*_m_, membrane potential.

Trial-to-trial *V*_m_ variability alters gain by changing the probability that a mean value of *V*_m_ will result in a spiking response but leaves PSP amplitude unaffected (Carandini 2004; Finn et al. 2007; Hansel and van Vreeswijk 2002). This therefore cannot account for the observed reduction in PSP amplitude under high-contrast stimulation but may still contribute to contrast gain control at the level of spiking responses. If this is the case, responses should be more variable under low-contrast stimulation, resulting in an increase in the gain of this relationship. We fitted a power law function to the mean membrane potential, ${\bar{V}}_{\text{m}}$, and mean spiking response across trials, $\bar{S}$, to investigate whether the gain of this relationship is indeed increased under low-contrast stimulation.

$$\bar{S}=k{\left[{\bar{V}}_{\text{m}}-{\bar{V}}_{\text{rest}}\right]}_{+}^{p}$$

where ${\bar{V}}_{\text{rest}}$ refers to the mean resting membrane potential of the neuron and the subscript “+” indicates half-wave rectification. The exponent *p* was fixed between contrast conditions but the gain factor *k* was allowed to vary. Some neurons showed an increase in gain under high-contrast stimulation (Fig. 10*A*), while others showed no change (Fig. 10*B*). No neurons showed a reduction in gain under high-contrast stimulation, as would be predicted if changes in membrane potential variance contributed to contrast gain control (Fig. 10*C*). As fluctuations in *V*_m_ are thought to underlie changes in this relationship, this finding suggests that *V*_m_ variability is not contrast dependent. We investigated this further by examining both the variance of *V*_m_ responses to high and low-contrast DRCs and the variability in noise-evoked PSP peak amplitude across trials (Fig. 10*D*). As predicted, no contrast-dependent changes in the standard deviation of *V*_m_, σ*_V_*_m_, were observed (low-contrast: 4.34 mV, high-contrast: 4.19 mV; signed-rank test, *P* = 0.426, Fig. 10*E*). Contrast-dependent changes were not observed in the standard deviation of peak response amplitude to the embedded noise stimuli either (low-contrast: 2.96 mV, high-contrast: 2.91 mV; signed-rank test, *P* = 0.426, Fig. 10*F*).

### Stimulus Contrast Does Not Modulate Input Conductance

**Fig. 11.**
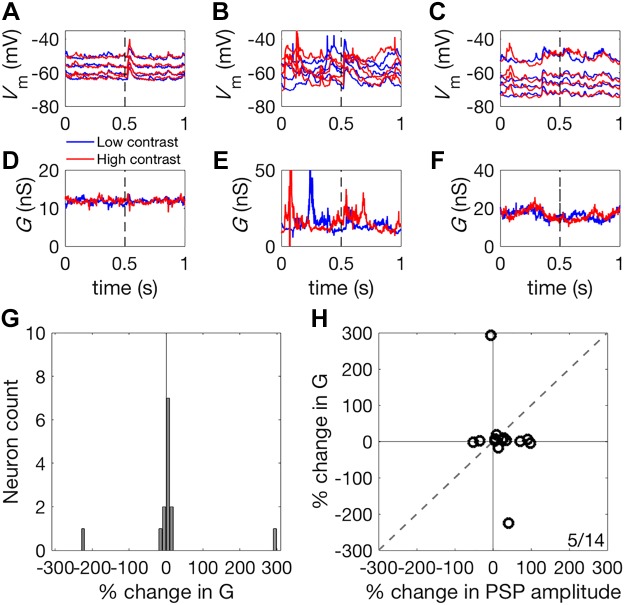
Input conductance during high- and low-contrast stimulation. *A*–*C*: membrane potential responses recorded under four different levels of current injection for three representative units, during high- (red) and low- (blue) contrast stimulation. Broken lines indicate the timing of noise stimulus presentation. *D*–*F*: gain, *G*, estimated for these same three units under high (red) and low (blue) contrast stimulation. *G*: histogram of % change in *G* in response to an approximate doubling of stimulus contrast. Gain, *G*, showed no systematic variation with stimulus contrast. *H*: scatter plot of contrast-dependent changes in PSP amplitude (abscissa) and *G* (ordinate). The change in *G* between contrast conditions was far smaller than changes that would be required to account for the contrast-dependent scaling of the evoked *V*_m_ response. The ratio in the corner indicates the number of neurons above the identity line, *y* = *x*, over the total number of neurons. PSP, postsynaptic potential; *V*_m_, membrane potential.

We next asked whether contrast-dependent changes in input conductance (*G*) could account for the reduction in PSP amplitude observed in our sample of 17 pyramidal neurons under high-contrast stimulation (Fig. 9*F*). To assess the contrast dependence of input conductance in the recorded neurons, *G* was estimated during stimulation with both high- and low-contrast DRCs (Fig. 11, *A*–*F*). Doubling the contrast had no systematic effect on *G* (median change in *G* = 2.37%; signed-rank test, *P* = 0.296, Fig. 11*G*). A change in *G* of −16.4% would have been required to account for the observed change in the slope of PSP amplitude from low to high-contrast stimulation. To account for the contrast-dependent scaling of PSPs, changes in *G* should be correlated with the observed changes in PSP amplitude. We found that contrast-dependent changes in *G* were unrelated to the contrast-dependent changes in the size of the PSPs (Fig. 11*H*). This was primarily attributable to the small magnitude of changes in *G* between contrast conditions, indicating that input conductance does not change with stimulus contrast.

## DISCUSSION

We investigated the biophysical basis of contrast gain control in mouse auditory cortex by examining the potential involvement of two mechanisms: PVI-mediated shunting inhibition and membrane potential variability. We first investigated whether PVIs are capable of modulating the gain of sensory-evoked responses in auditory cortex, as they are in V1 (Atallah et al. 2012; Wilson et al. 2012). We did this by optogenetically stimulating or suppressing PVI activity during auditory stimulation while recording MUA from the auditory cortex of anesthetized mice.

Changes in neural responses between conditions can involve multiplicative/divisive changes in gain or subtractive/additive changes in the offset of responses. Arch-mediated suppression of PVI activity resulted in a significant increase in the overall gain of auditory cortical responses, in addition to a small subtractive change in offset. However, the magnitude of this gain change was similar between contrast conditions, resulting in no effect of PVI suppression on contrast-dependent gain changes, i.e., contrast gain control was unaffected by suppressing the activity of PVIs. ChR2-mediated activation of PVIs similarly altered the overall gain and offset of auditory cortical responses. In this case, however, when compared across contrast conditions, the differences were still significant, indicating an effect of PVI activation on contrast gain control. Both optogenetic manipulations had appreciable effects on overall gain (5%) of cortical neurons but smaller effects on contrast gain control (~1%). This suggests that PVI activity modulates the gain of auditory cortical responses, but not in the contrast-specific way that would be required for PVIs to be the primary mechanism for contrast gain control in auditory cortex.

Activation of PVIs in mouse auditory cortex with ChR2 has previously been found to produce a mixture of divisive and subtractive effects on sensory evoked responses (Seybold et al. 2015). In keeping with this observation, we found that while PVI activation reduces gain it also produces a large reduction in offset. Previous conflicting results regarding the subtractive or divisive nature of PVI activity in V1 have been found to be attributable to unnatural levels of interneuron activation, which artificially suppresses responses below threshold, changing divisive changes in gain into subtractive changes (Atallah et al. 2012, 2014; Lee et al. 2012, 2014; Wilson et al. 2012). To avoid this concern, we aimed to induce modest activation of PVIs but nonetheless still observed both divisive and subtractive effects.

The lack of contrast-dependent divisive changes in auditory cortex response gain during manipulation of PVI activity raises the possibility that the effects of activating these interneurons may differ between cortical areas. Another potential explanation is that firing rates in auditory cortex in response to DRCs are typically much lower than V1 responses to the drifting grating stimuli typically used in these experiments. Smaller levels of suppression may therefore be required to produce subtractive effects due to interactions with spike threshold during a low firing rate regime. If this is the case, PVI activation might be expected to have a subtractive effect in V1 during stimulation with natural scenes, which typically evoke lower firing rates than gratings (Gallant et al. 1998).

Seybold et al. (2015) argue that the true effects of inhibition provided by distinct neuronal subpopulations could be masked when these neurons are activated artificially, due to recurrent connectivity of cortical networks and to interactions with spike threshold. In keeping with this, optogenetic activation inevitably introduces a somewhat artificial, synchronous activation into the activity patterns of the stimulated population at light onset, which may significantly disrupt normal network operation. Controlled optogenetic suppression may interfere less with typical network operation by leaving the statistical structure of spiking activity largely unchanged, as it does not introduce new spikes with unnatural correlations. Arch-mediated PVI suppression therefore represents a more reliable method for interrogating the role of interneuron inhibition in gain control than PVI activation. Because PVI suppression had no effect on contrast gain control in auditory cortex, despite producing marked changes in response gain at each contrast, it seems unlikely that these inhibitory interneurons contribute mechanistically to this form of adaptation.

We obtained additional evidence for this by investigating whether PVIs are recruited during high-contrast stimulation in a manner that would allow them to mediate contrast gain control in auditory cortex. By using virally targeted expression of ChR2 to optically tag PVIs, we found that the majority (65%) of the PVIs in auditory cortex which were identified by this method did not significantly increase their activity with increasing stimulus contrast. This is in contrast to V1, where PVI firing rates have been found to be generally tightly linked to stimulus contrast (Atallah et al. 2012). This further suggests that the mechanisms underlying contrast gain control, or at least the contribution made to that process by PVIs, may differ between sensory areas or modalities.

Nevertheless, cortical interneurons have been implicated in different aspects of auditory processing, including adaptation to stimulus statistics (Blackwell and Geffen 2017). This has previously been examined in the context of stimulus-specific adaptation. Optogenetic suppression of PVIs has been shown to induce a nonselective increase in excitatory cortical responses, whereas somatostatin interneuron suppression affects responses to frequent rather than rare tones (Natan et al. 2015). Along with other evidence, this suggests different roles for these two types of cortical interneuron in context-dependent sound processing (Phillips et al. 2017) and a specific role for somatostatin interneurons in adaptation to repeated sounds (Kato et al. 2015; Natan et al. 2015). Although we have not examined the contribution of the latter to contrast gain control, our results are consistent with these studies in suggesting that PVIs are the not the key cell type in the auditory cortex that is responsible for context-specific gain modulation.

Contrast gain control in auditory cortex has previously been observed at the level of spiking responses (Rabinowitz et al. 2011) but has not been documented at the level of the membrane potential. We observed that membrane potential responses exhibited divisive scaling in amplitude during high-contrast compared with low-contrast stimulation. This is consistent with the hypothesis that shunting inhibition might be a key mechanism in auditory cortex contrast gain control. At the same time, this finding argues against the membrane potential variance hypothesis (Finn et al. 2007), because it indicates that contrast-dependent membrane potential fluctuations alone cannot account for contrast gain control in auditory cortex. We also compared the standard deviation of evoked PSP amplitudes between contrast conditions and found no systematic difference, which provides further evidence against the membrane potential variance hypothesis. In keeping with this, the gain of the relationship between mean membrane potential and firing rate was not reduced during high-contrast stimulation in auditory cortex neurons, unlike in V1 where this has been found to be the case (Finn et al. 2007).

Shunting inhibition is thought to modulate gain by increasing the input conductance of neurons to divisively scale PSP amplitude (Carandini and Heeger 1994; Carandini et al. 1997). We found, however, that input conductance was not significantly modulated by stimulus contrast. In V1, input conductance can increase by up to 300% during the presentation of a high-contrast grating with reference to baseline input conductance (Anderson et al. 2000). Here we observed no such effect, suggesting that, in auditory cortex, shunting inhibition does not contribute to contrast gain control. Our recordings were performed across the depth of cortex, and it is possible that shunting inhibition is recruited by thalamic inputs and may therefore be confined to neurons in layer 4. Targeted recordings of input conductance in layer 4 neurons during stimulation with stimuli of different contrasts will be necessary to address this issue.

In summary, our findings provide evidence that contrast gain control in auditory cortex is not implemented by shunting inhibition from PVIs. Short-term synaptic depression at the thalamocortical synapse is believed to contribute to contrast gain control in V1 and may also do so in auditory cortex (Banitt et al. 2007; Carandini et al. 2002). Recent evidence has demonstrated that contrast gain control is also exhibited by neurons in the mouse inferior colliculus and medial geniculate body, indicating that this is not an emergent property of auditory cortex (Lohse et al. 2020). Nevertheless, the time constants of contrast adaptation are longer in the auditory cortex than at subcortical levels (Lohse et al. 2020; Rabinowitz et al. 2013), so it is likely that local processing contributes to these computations at each processing level. Further characterization of the circuits and mechanisms underlying canonical computations such as contrast gain control across sensory modalities holds the promise of not only providing insight into the specific system being studied but also into fundamental questions regarding the relationship between mechanistic and computational levels of understanding in neuroscience.

## GRANTS

This work was supported by the Wellcome Trust through Principal Research Fellowship Grants WT076508AIA and WT108369/Z/2015/Z (to A. J. King) and a 4-yr studentship (096588/Z/11/Z; to J. E. Cooke).

## DISCLOSURES

No conflicts of interest, financial or otherwise, are declared by the authors.

## AUTHOR CONTRIBUTIONS

J.E.C., E.O.M., A.J.K., J.W.H.S., and B.D.W. conceived and designed research; J.E.C. and M.K. performed experiments; J.E.C., J.W.H.S., and B.D.W. analyzed data; J.E.C., M.K., E.O.M., A.J.K., J.W.H.S., and B.D.W. interpreted results of experiments; J.E.C. prepared figures; J.E.C. drafted manuscript; J.E.C., M.K., E.O.M., A.J.K., J.W.H.S., and B.D.W. edited and revised manuscript; J.E.C., M.K., E.O.M., A.J.K., J.W.H.S., and B.D.W. approved final version of manuscript.
